# Synaptic tagging and capture: a bridge from molecular to behaviour

**DOI:** 10.1186/1471-2202-12-S1-P122

**Published:** 2011-07-18

**Authors:** Lorric Ziegler, Wulfram Gerstner

**Affiliations:** 1 School of Computer and Communication Sciences and Brain Mind Institute, Ecole Polytechnique Federale de Lausanne, 1015 Lausanne EPFL, Switzerland

## 

Hippocampal plasticity is now widely accepted as being the substrate for episodic memory formation. More specifically, several studies in area CA1 of the hippocampus support the Synaptic Tagging and Capture hypothesis (STC) which describes the induction as well as the consolidation phases of synaptic Long-Term Potentiation/Depression (LTP/D) (for a review see [1]). In this framework, initial changes in the synaptic weights require an external signal, sometimes called 3^rd^ factor, in order to persist over time. This maintenance signal, comparable to the reward of reinforcement learning theory, is non local and leads to associative effects, i.e. synaptic modifications induced by a weak LTP/D event can be rescued by a strong event happening closely in time. Recently, new experimental paradigms corresponding to a behavioural analog of STC have been developed [2,3]. In these setups, Long-Term Memory (LTM) of rodents is probed in different contexts in order to determine how weak versus strong stimuli, as well as open field exploration, influence the lifetime of the memory trace. In the case of novel environment exploration, similar associative effects can be observed as in CA1 slices measurements, where an independent but behaviourally relevant stimulus can help maintain an otherwise decaying memory trace.

We propose a simple model network of the medial temporal lobe to bridge the two concepts (LTP/D and LTM) within the framework of STC. The model consists of rate nodes and connectivity patterns inspired from the hippocampus and the lateral amygdala. In order to simulate initial storage and longer temporal evolution of spatial or emotional memory, we implement hippocampal plasticity with a rate version of the Tag-Trigger-Consolidation (TagTriC) model [4], developed to reproduce induction and maintenance of LTP/D in CA1. Finally animal behaviour is derived from elementary statistics of decision units within the network. Simulation results show that the functional properties of TagTriC transpose to this behavioural paradigm. Moreover, the model was able to explain an interference observed when the rescuing event, e.g. open field exploration, occurs too close to the memory encoding event. Responsible for this interference is a resetting of the plastic modifications induced within the same network parts when active consecutively in different contexts. This interaction unfolds naturally from our modified TagTriC consistent with tag resetting *in vitro*[5].

This work intends to make a first step towards integration of different levels, functional plasticity and behaviour, of a more general theory of memory. It supports the tagging and capture hypothesis as a basic building block for episodic memory.
